# Implementation of Communication Disability Documentation and Accommodations in Primary Care: A Pragmatic Type III Hybrid Implementation-Effectiveness Trial Protocol

**DOI:** 10.1044/2026_JSLHR-26-00080

**Published:** 2026-10-02

**Authors:** Jennifer Oshita, Christina Studts, Carol Haywood, Alicia Wong, Jennifer Duffecy, Megan A. Morris

**Affiliations:** a Department of Rehabilitation Medicine, NYU Grossman School of Medicine, New York; b Department of Pediatrics, University of Colorado Anschutz Medical Campus, Aurora; c Department of Medical Social Sciences, Northwestern Feinberg School of Medicine, Chicago, IL; d Denver Health, CO; e Department of Psychology, University of Illinois Chicago

## Abstract

**Purpose:**

Routine collection of patients' communication disability (CD) status is instrumental for monitoring and addressing health disparities, including the provision of disability accommodations. Few health systems routinely collect patients' CD status. This article describes the study protocol for the “Documentation of Communication Disability Collection and Accommodations in Primary Care Settings” (DECA) study, a pragmatic Type III hybrid implementation-effectiveness trial evaluating an implementation guide and practice facilitation on the adoption of CD status documentation. We additionally evaluate the study's pragmatic design using Pragmatic Explanatory Continuum Indicator Summary-2–Provider Strategies (PRECIS-2-PS).

**Method:**

The intervention includes documenting CD status and accommodation needs in the electronic health record (EHR) and providing requested accommodations. The implementation strategies are a tailored implementation guide and a practice facilitator providing implementation planning, problem solving, and quality monitoring. This quasi-experimental, stepped-wedge design includes 10 clinics across five health systems. The study employs a concurrent mixed-methods design. The primary outcome is Reach, operationalized as the proportion of patients with clinic visits during the study period who have CD status documented in the EHR. Secondary outcomes include the percentage of patients with accommodations documented in the EHR and the qualitative evaluation of adoption, implementation, and maintenance. Using PRECIS-2-PS domains, we rated the DECA study design elements as moderately to highly pragmatic, with design trade-offs made to balance real-world applicability with scientific rigor.

**Conclusion:**

The DECA study will offer pragmatic evidence on the implementation effectiveness of an implementation guide and practice facilitation supporting health systems in identifying which patients have a CD and what accommodations they need.

**Supplemental Material:**

https://doi.org/10.23641/asha.33967966

The discipline of communication sciences and disorders (CSD) strives to make “effective communication, a human right, accessible and achievable for all,” not only through rehabilitating communication functioning but also through supporting participation in activities and addressing environments of communication (American Speech-Language-Hearing Association, 2016; O'Halloran et al., 2008; World Health Organization, 2002). These environments include high-stakes health care settings, where people with communication disabilities (CDs) in speech, hearing, language, and voice experience significant disparities in outcomes. Examples of these disparities include experiencing more adverse events when hospitalized (Bartlett et al., 2008), greater difficulty finding a primary care provider, greater dissatisfaction with their care (Reed et al., 2019, 2021), more perinatal complications (Mitra et al., 2020), and higher rates of emergency department use (James et al., 2022; Lauer et al., 2021; Stransky et al., 2018), compared to adults without CDs.

People with CDs report that it is often the environment of health care that contributes to ineffective communication. Health care teams are unprepared to communicate with them (Morris, Wong, et al., 2021) and do not communicate in ways that they prefer and are effective (Iezzoni et al., 2004; Morris et al., 2015). People with CDs also report insufficient time to communicate and inappropriate assumptions made about their competence (Morris et al., 2013). Primary care providers corroborate these experiences—they feel unable to collect an adequate history or understand patient complaints and struggle to use supportive communication strategies (Agaronnik, Campbell, et al., 2019; Agaronnik, Pendo, et al., 2019; Burns et al., 2015; Iezzoni et al., 2023; Zivianni et al., 2004). Providers express a desire for disability information to better care for their patients (Morris, Lockhart, et al., 2021; Mudrick et al., 2020).

## Research-to-Practice Gap: Documenting Disability Status in the Electronic Health Record and Provision of Accommodations

Addressing the environment of communication in health care for people with CDs is a system-level approach to achieving effective communication (Simmons-Mackie, 2013). It has been advocated for in the literature and begins with documentation of CD status and accommodation needs in the electronic health record (EHR) and subsequently providing documented accommodations. We define accommodations as modifications and supports essential to access safe, effective, and equitable health care services for people with CDs. Both documenting disability status and providing accommodations during health care are prioritized as urgent research needs by the disability community (Morris, Lockhart, et al., 2021; Morris, Schliep, et al., 2017). Documentation of disability status enables all health care team members' access to this critical information in the EHR. At a population level, these data are central to monitoring and addressing health care disparities (Centers for Medicare & Medicaid Services, 2022, 2024; Patient Protection and Affordable Care Act, 2010). It is also mandated by Section 4302 of the Patient Protection and Affordable Care Act (2010) and endorsed by health organizations such as the National Academy of Medicine, the Centers for Medicare & Medicaid Services, the Joint Commission, and the National Committee for Quality Assurance (Centers for Medicare & Medicaid Services, 2024; Institute of Medicine, 2013, 2014; National Center for Quality Assurance, 2026; The Joint Commission, 2025). Routine provision of accommodations is also a legally mandated part of health care provision Americans with Disabilities Act of 1990, as Amended (2009; United States Department of Justice Civil Rights Division, 2021) and endorsed as best practice (The Joint Commission, 2010, 2025).

Unfortunately, most health systems, hospitals, and clinics do not routinely collect CD status data or provide accommodations (National Academies of Sciences, Engineering, and Medicine, 2024). Health systems report a lack of guidance, and standardized processes are major barriers to implementation (Morris et al., 2024; Oshita et al., 2024; Sarmiento et al., 2025). Experience with collection of other demographic data (e.g., race, sexual orientation and gender identity) and response to social needs demonstrates that health systems require targeted support and implementation strategies to achieve routine, high-quality data collection and response workflows (Gold et al., 2017, 2018; Gruß et al., 2021; Holt et al., 2021; Lee et al., 2016; Palaniappan et al., 2009).

We designed the following study to improve the environment of communication in health care for people with CDs through the implementation of CD status documentation and the provision of accommodations. As improving the environment of communication in health care is relevant beyond the walls of CSD and most efficient when implemented at a system level (Simmons-Mackie, 2013; Simmons-Mackie et al., 2007; The Joint Commission, 2010), we focused on primary care. Primary care is the health care home for most adults, thus providing consistent opportunities for system-level documentation and encounter-level accommodation provision.

## Pragmatic Research

Developing evidence on how to implement the collection of CD status in the EHR and then use the information to provide accommodations requires conducting the study in real-world settings rather than highly controlled research environments. Implementation strategies must be tested in the contexts where they will ultimately be used, as local factors, workflows, and resources fundamentally shape what strategies will be feasible and effective. As such, we are conducting a pragmatic trial. Pragmatic research “seeks to understand if innovations work in real-world contexts that closely reflect the settings in which the innovation is intended to be used” (Norton et al., 2021). To accomplish this, pragmatic research aims to enhance external validity while still balancing internal validity considerations. In contrast, explanatory research seeks to understand whether an intervention works under ideal conditions (Loudon et al., 2015). Explanatory studies maximize internal validity through stringent inclusion criteria and rigid protocol delivery (e.g., skilled clinicians, research assistants administering intervention, intense follow-up) but have been critiqued for their limited real-world applicability (Glasgow, 2013; Glasgow et al., 2006; Holtrop & Glasgow, 2020). For implementation science questions, which focus on “how” to integrate evidence-based practices into routine care, pragmatic approaches are particularly appropriate as they prioritize external validity and real-world applicability (Brown et al., 2017; Curran et al., 2022).

In this article, we present the research protocol for “Implementation of Communication Disability Collection and Accommodations in Primary Care Settings” (the DECA study), a pragmatic Type III hybrid implementation-effectiveness trial. We highlight the decisions and trade-offs encountered to improve the interpretability of the study's findings and to provide practical guidance for other researchers interested in conducting pragmatic implementation trials. We organize the article by first describing the study objectives, the intervention and implementation strategies, and the study design. We then present our outcomes and analysis plan. Finally, we evaluate the study using the Pragmatic Explanatory Continuum Indicator Summary-2–Provider Strategies (PRECIS-2-PS) framework, a validated tool for assessing the pragmatic nature of implementation trials (Norton et al., 2021). The following paper adheres to the reporting guideline: Standards for Reporting Implementation Studies, which is EQUATOR Network approved (Pinnock et al., 2017; see Supplemental Material S1 for the completed checklist).

## Study Objectives

The DECA study aims to provide evidence on how to integrate the routine documentation of CD status and accommodation needs in the EHR and support the provision of accommodations in primary care, an essential part of an effective communication environment in health care for people with CDs. We will evaluate implementation strategies to increase the documentation and provision of accommodations, with the overarching goal of promoting equitable and accessible primary care for persons with CDs. Although disability documentation policies apply to all disability types, this study intentionally focuses on CD as a feasible starting population for whom the intervention would have a significant impact.

The DECA study is a Type III hybrid implementation-effectiveness study, meaning that the main goal is to evaluate the implementation strategies, with a secondary goal of evaluating its effectiveness (Curran et al., 2012). While typically appropriate for interventions with established effectiveness, there is growing recognition that this study design is also appropriate for evaluating policy implementation (Purtle et al., 2023). Given multiple legal and policy requirements for documenting disability status and providing accommodations for people with CD, it is appropriate to focus on implementing these policies. Effectiveness of the accommodations provided will be an exploratory aim. This study was approved by the institutional review boards at NYU Langone Health. The study is funded by the National Institutes of Health R01 (R01DC020188).

### The Policy-Based Intervention: CD Documentation and Accommodation Provision

The intervention consists of two components: (a) documenting CD status and accommodation needs in the EHR and (b) providing requested accommodations. Documentation of CD status includes two evidence-based CD questions combined: (a) “Are you deaf or do you have serious difficulty hearing?” and (b) “In your usual language, do you have difficulty understanding or being understood?” (Morris, Lagu, et al., 2017; Washington Group on Disability Statistics, 2020). In the health care setting, these demographic questions are self-reported and do not provide or relate to a clinical diagnosis but rather an identity status and an accommodation need. As such, the questions can also be asked by anyone involved in coordinating or delivering accommodations (e.g., registrars and schedulers, in addition to clinicians).

Clinics have flexibility in how they collect this information (e.g., patients completing a form in the waiting room, collection through the patient portal, or medical assistants asking during encounters). All clinics must enter the information into the patient's EHR. While the study focuses on CD, clinics may also choose to collect information on other disabilities and accommodations.

To ensure that all participating clinics have access to the same communication accommodations, the accommodations being documented and provided in this study are limited to hearing amplifiers and communication boards. We provide each clinic $400 to purchase hearing amplification devices and communication boards. Additionally, each clinic receives $2,000 to offset the costs of participating in the study. Clinics have flexibility to develop processes connecting the documentation and provision of accommodations in ways that fit their local workflows and clinic structure. The implementation guide supports clinics in developing these processes regardless of their chosen approach.

### Implementation Strategies

#### Implementation Guide

We first developed an implementation guide for documenting disability status and accommodation needs and providing accommodations using a process called implementation mapping (Fernandez et al., 2019; Oshita et al., 2025). This systematic stakeholder-driven process involved a multistakeholder group, including disability experts, researchers, health system stakeholders, and people with disabilities who collaboratively identified barriers to implementation and relevant strategies to address these barriers. The resulting implementation guide provides recommendations on workflow and EHR build components for disability and accommodation documentation. It also includes planning tools such as a needs assessment worksheet, implementation planning worksheet, sample patient scripts explaining reasons for disability documentation, disability status question prompts, frequently asked questions, and clinic and patient education materials.

#### Practice Facilitation

To support clinics in using the implementation guide, our implementation strategy is practice facilitation. Practice facilitation engages clinics in interactive problem solving, supporting the adaptation of the intervention to respond to local barriers, and monitoring progress (Cole et al., 2024; Kilbourne et al., 2023). Practice facilitators are skilled in addressing EHR and data challenges in the implementation of new approaches to care (Hemler et al., 2018). Multiple studies demonstrate that practice facilitation improves the implementation of advanced primary care models, making it an evidence-based choice for supporting the integration of new documentation and care processes (Gold et al., 2017, 2018).

A single skilled, external practice facilitator will work with all 10 participating primary care clinics. The practice facilitator has 15 years of experience working in health care quality and practice transformation. She has worked with over 100 clinics around the country as a practice facilitator to implement innovations in the primary care setting. She is supported by and works within the Practice Transformation Program at the University of Colorado Anschutz.

The facilitator will virtually meet with each clinic's implementation team once monthly for 9 months. During these meetings, the practice facilitator will use the implementation guide to (a) provide technical assistance, (b) assist in developing implementation processes and problem-solving challenges that arise, and (c) provide direction on using tools for quality monitoring and promoting local adaptations.

Each clinic will identify a two- to three-member implementation team who will participate in monthly facilitation sessions and lead local implementation efforts. Implementation team members may include practice managers, physicians, medical directors, medical assistant leads, or quality improvement clinic leads. The implementation team is responsible for (a) training health care team members on CD documentation and accommodation provision, (b) assessing the clinic for readiness and identifying barriers, (c) conducting a local needs assessment around disability documentation and accommodations, (d) developing a formal implementation plan tailored to their clinic's workflows and resources, (e) leading implementation efforts, and (f) monitoring their clinic's progress for internal reporting.

### Study Sites

We are conducting the study at 10 primary care clinics across five health systems (two clinics per health system): UCHealth (affiliated with University of Colorado Anschutz Medical Campus), Denver Health, Northwestern Medicine (affiliated with Northwestern University), UI Health (affiliated with the University of Illinois Chicago), and NYU Langone Health. All participating sites use the Epic EHR system and have electronic data warehouses that enable EHR data extraction for outcomes. The sites represent diverse primary care settings, including six safety-net clinics, two clinics that specialize in caring for patients with disabilities, and a geriatrics clinic.

### Study Design

The DECA study uses a cluster design in which each primary care clinic serves as the unit of analysis (Brown et al., 2017). We selected a cluster design because we aim to change the behavior of clinic staff and clinicians rather than individual patients. Additionally, contextual factors within each primary care clinic (e.g., availability of accommodations, workflow processes, clinic culture) influence the entire care team, making it infeasible to provide the intervention to only some staff members without contamination.

We are implementing a stepped wedge design in which all participating clinics receive practice facilitation at some point (Brown et al., 2017; NIH Pragmatic Trials Collaboratory, n.d.). In a stepped wedge design, clinics transition from a control condition (baseline period) to the intervention condition (practice facilitation period), with each clinic serving as its own control. A key benefit of the stepped wedge design is that all clinics receive the intervention, which increases leadership willingness to participate in the study (Brown et al., 2017). The within-clinic comparison in the stepped wedge design also provides greater statistical efficiency compared with parallel designs, requiring fewer total clusters to achieve equivalent power (Hemming et al., 2015).

## Evolution From Randomized to Quasi-Experimental Design

We originally planned a randomized stepped wedge design in which each clinic would be randomly assigned to begin the practice facilitation intervention at specific time points. However, two major external contextual developments required a shift to a quasi-experimental design in which clinics determine when they begin practice facilitation.

### Office of the National Coordinator EHR Standardization

In 2023, 1 year after the grant was funded, the Office of the National Coordinator for Health Information Technology released recommendations for a standard disability data element for all EHRs (Assistant Secretary for Technology Policy, 2023). Consequently, Epic (the EHR vendor used by all participating sites) announced plans to include disability as a data element on their platform and began developing a standardized build for all customers to collect disability status and accommodation needs. Many sites planned to develop their own EHR build as part of their study participation. However, with the impending Epic release, they became hesitant to invest resources in developing their own system. Unfortunately, Epic did not release a standardized build within our study timeline, requiring sites to ultimately create their own builds for collecting disability status and accommodation needs, delaying when clinics could begin the intervention.

### Joint Commission Health Equity Certification

Also in 2023, the Joint Commission released a voluntary Health Equity certification (now called Excellent Health Outcomes for All certification; The Joint Commission, 2023). To qualify, a health system must demonstrate a routine collection of patients' disability status. Several participating sites decided to pursue this certification and so were unwilling to be randomly assigned to intervention start times.

Given these contextual factors, the original randomized stepped wedge design became incompatible with sites' needs. Allowing the clinics to begin the practice facilitation when they were ready prioritizes external validity by evaluating implementation in sites representative of real-world adopters—those whose leadership proactively chooses to implement disability documentation initiatives in response to changing policies and accreditation requirements. However, this required moving to a quasi-experimental design that reduces internal validity through potential selection bias and confounding by time.

## Final Quasi-Experimental Stepped Wedge Design

Sites retain flexibility to determine when they transition from baseline to intervention periods, based on EHR build completion and clinic readiness. Clinic start times will occur across a 12-month period, still allowing for a stepped implementation pattern. Each clinic contributes 3 months of baseline EHR data (before practice facilitation), 9 months of intervention data (during practice facilitation), and 3 months of maintenance data (after practice facilitation ends; see Figure 1). The entire study period spans 15 months for each clinic. The intervention period starts the month that a clinic has their first practice facilitation meeting. Baseline data, which is the EHR data, will be the 3 months immediately prior to the intervention period.

**Figure 1.**
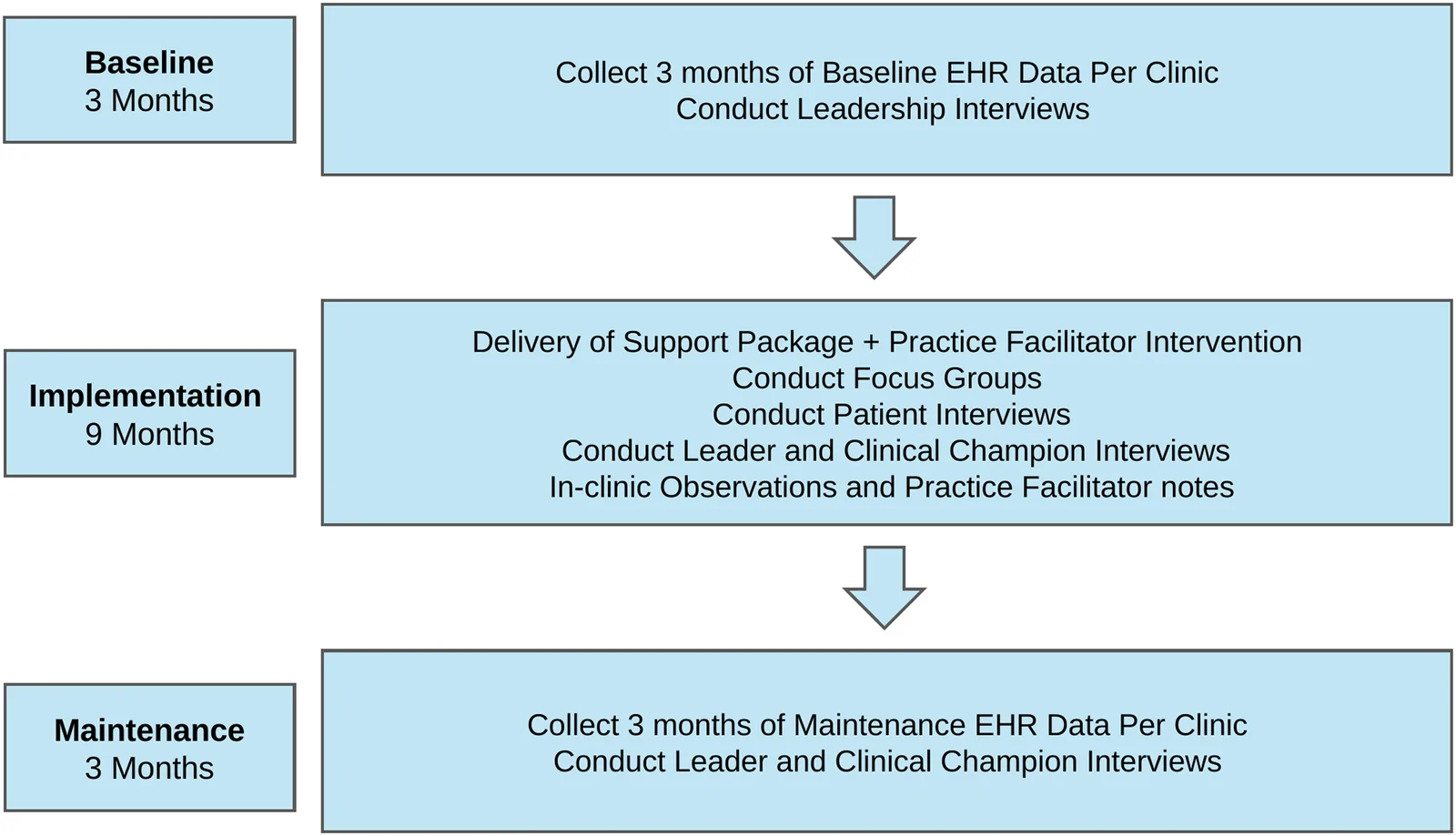
DECA study flow diagram. DECA = Documentation of Communication Disability Collection and Accommodations in Primary Care Settings; EHR = electronic health record.

The stepped design provides important advantages even without randomization. First, it allows accounting for secular trends (changes over time unrelated to the intervention) by comparing clinics in the baseline period to clinics in the intervention period at overlapping calendar time points. Second, staggered implementation reduces burden on the practice facilitator and research team, allowing more intensive support to each clinic during their intervention period. Third, later-starting clinics benefit from lessons learned from clinics who began earlier.

## Outcomes

Given that the DECA study is a Hybrid Type III implementation-effectiveness study, we organize outcomes using the RE-AIM implementation framework, which includes five domains: Reach, Effectiveness, Adoption, Implementation, and Maintenance (Glasgow et al., 1999). Reach is the proportion and representativeness of individuals (patients) who participate in or are exposed to the intervention. Effectiveness is the impact of the intervention on outcomes. Adoption is the proportion and representativeness of settings and staff who implement the intervention. Implementation is the extent to which the intervention is delivered as intended and the adaptations made to fit local contexts. Maintenance is the extent to which the intervention becomes (or plans to become) part of routine practice. (See Table 1 for a list of the outcomes by the RE-AIM framework.)

**Table 1.** Outcomes and data sources using the RE-AIM framework.

Variable	Documenting disability outcomes	Documenting accommodation needs and providing accommodations outcomes	Data source
Reach	The % of patients with disability status documented in the EHR^a^	For patients with a CD, % of patient with accommodation needs documented in the EHR	1. Electronic data warehouse
Effectiveness	N/A	1. Providers' rating on the effectiveness of the accommodation 2. Patients' perceptions of whether the accommodation improved communication	1. Provider survey 2. Patient interviews
Adoption	Perceptions of adoption	Whether patients' requested accommodation was used during the clinical encounter	1. Leadership interviews 2. Staff and providers focus groups 3. Provider survey
Implementation	1, Whether the staff consistently asked and recorded disability status 2. Adaptations to the workflow and planned implementation	1. Whether the staff consistently provided an documented accommodation 2. Adaptations to the workflow and planned implementation	1. Practice observations 2. Practice facilitator's notes 3. Leadership interviews 4. Staff and providers focus groups 5. Patient interviews 6. Provider survey
Maintenance	Reach and implementation outcomes during maintenance period	Reach and implementation outcomes during maintenance period	1. Electronic data warehouse 2. Interviews with leadership

*Note.* EHR = electronic health record; CD = communication disability; N/A = not applicable.

a Primary outcome.

We will use a concurrent mixed-methods design, collecting both quantitative and qualitative data throughout the study (Creswell & Clark, 2017). Mixed-methods designs are common in implementation science and provide not only critical information about the outcomes but also the contextual factors contributing to these outcomes (Palinkas & Cooper, 2017). We will collect both qualitative and quantitative data concurrently and will integrate the findings during analysis to identify patterns and insights from multiple data sources.

### Primary Outcome

#### Reach

The primary goal is increasing the collection of patients' CD status in the EHR in the primary care setting. As such, our primary outcome is Reach, operationalized as the proportion of all patients who have a visit in the clinic during the study period and have their CD status documented in the EHR. A positive outcome includes any response in the CD fields: “no disability,” “communication disability,” “hearing disability,” “decline to answer,” or “other.” A negative outcome is the CD fields being left blank. We consider “decline to answer” a positive outcome because it indicates that the question was asked and the patient's response was recorded, which fulfills the goal of attempting to collect this information systematically. All adult patients (18 years and older) who are seen in each clinic are eligible to have their disability status documented, regardless of visit type. For the primary outcome analysis, we will only count a patient's first visit during the study period. As an exploratory analysis, we will include patients' follow-up visits if they had the field left blank, to understand whether adoption increased over time. This outcome will be collected through each site's electronic data warehouse, which is a local data repository for all EHR data. We estimate that approximately 600 patients will be seen in each clinic each month. With 15 months of data collection per clinic and 10 clinics, this equals approximately 90,000 eligible patients.

### Secondary Outcomes

#### Reach—Accommodation Needs Documentation

For patients who have a CD documented in the EHR, we will measure the percentage of patients who have the accommodation field completed. This includes the documentation of specific accommodation requests and the documentation of instanced where no accommodation is needed. This outcome will be collected through the EHR.

#### Adoption

We will assess adoption through qualitative interviews with clinic leadership conducted at three time points: prior to the intervention start, at the midpoint, and during the maintenance period. We will conduct approximately 90 interviews total (3 leaders per clinic × 10 clinics × 3 time points). We will also conduct focus groups with staff and clinicians (two focus groups per clinic for 20 focus groups). We will develop semistructured interview and focus group guides that probe participants' perceptions of adoption, the implementation and sustainability infrastructure, the intervention itself (documentation and accommodations), and factors that influence adoption and implementation.

#### Implementation

We will assess implementation through multiple data sources. Implementation fidelity (whether staff consistently asked and recorded CD status and whether staff provided accommodations when a need is documented in the EHR) will be understood through the leadership interviews and staff and clinician focus groups. Additionally, research staff will conduct up to three practice observations during clinics' intervention period. The observations will last no more than half a day and will not include observations of clinical encounters. Instead, the observations will document organizational workflows and clinic structures and environment. For example, the research assistant will observe the process for how patients check in and are roomed. A staff member will walk the research assistant through the process of documenting the patient's disability and accommodation information in the chart and the workflow for providing the accommodation. Additionally, they will observe where the accommodations are stored and how accessible they are to all team members and any patient-facing materials related to documenting disability and availability of accommodations. Finally, they will document the accessibility features of the clinic (e.g., availability of wheelchair seating in waiting room, availability of quiet spaces for patients to wait) to understand the culture of accessibility in the clinic.

Using a standardized form, the practice facilitator will also document all meetings, including clinic progress, barriers, and adaptations made during the implementation process through meeting notes taken during facilitation sessions. These notes will be monitored to ensure fidelity. We will also assess implementation through patient interviews and provider surveys below.

#### Maintenance

We will evaluate maintenance through collecting EHR data during the 3-month maintenance period following practice facilitation and through the final leadership interviews conducted during the maintenance period.

### Exploratory Outcome

#### Effectiveness

As an exploratory outcome, we will assess the effectiveness of communication accommodations. First, we will survey providers after their visits with patients who have accommodation requests documented. The survey will ask providers if the accommodation was provided and, if so, to rate the effectiveness of the accommodation in facilitating communication. We aim to collect approximately three provider surveys per clinic per month during the 9-month intervention period, for up to 270 surveys. Second, we will conduct patient interviews to understand patients' perceptions of whether the accommodation improved communication. We will conduct approximately five patient interviews per clinic for 50 in total. This sample size was chosen based on standard qualitative research methods, which suggest that data saturation can be reached with as few as five participants in a subgroup (Creswell & Poth, 2016). This approach ensures both robust data collection and feasibility within the study's scope and resources. During interviews, we will ask patients whether their documented accommodation was provided during their visit and their perceptions of its effectiveness. Additionally, patients will be asked about their experience with being asked about their CD and accommodation needs. Recruitment approaches will vary by clinic based on local feasibility and workflows.

Effectiveness was exploratory rather than a secondary outcome, as health care teams are federally mandated to provide accommodations to patients with disabilities when requested, regardless of measured effectiveness. However, stakeholders identified understanding accommodation effectiveness as important for clinician and leadership buy-in for initiatives to document disability and provide accommodations (Morris, Lockhart, et al., 2021).

## Contextual Factors

We will additionally systematically collect information on contextual factors that could influence the study implementation and collection of CD status and accommodation needs. First, during the initial interviews with clinic leadership, we will collect information on institutional and clinic context (e.g., current initiatives aimed at caring for patients with disabilities, recent or planned organizational changes, other quality improvement efforts). Second, the practice facilitator will note contextual factors in meeting notes (e.g., clinic relocations, staffing changes, implementation of other initiatives during the intervention period). Finally, the study team will systematically document broader contextual factors that may influence the study (e.g., changes in EHR functionality at the site, changes in federal support for equity initiatives, other clinical areas within the health system initiating the collection of disability status). These data will be integrated into the qualitative data analysis.

## Analysis Plan

We expect 600 patients to visit each clinic per month, for approximately 6,000 patients per month across all clinics. Over the 15-month data collection period, each clinic will see approximately 9,000 patients. With 10 clinics, we expect a total sample size of 90,000 patients. For the primary outcome, assuming an intraclass correlation coefficient of 5%, this sample size will provide > 90% power to detect an effect size (difference in proportions) of 0.01 between baseline and intervention conditions. This assumes a baseline disability field completion rate of 15% based on preliminary data (Morris, Hamer, et al., 2021).

### Quantitative Analysis

We will compute descriptive statistics for baseline patient and clinic characteristics, examining differences between the baseline and intervention periods using chi-square tests and *t* tests. For survey-based outcomes (provider surveys), we will also compare responders versus nonresponders to assess potential response bias. Patient-level factors will be included in multiple regression models if they are imbalanced between study periods at baseline and if the study team determines they are clinically meaningful variables.

We will use binomial generalized linear mixed models to evaluate the primary outcome, a binary outcome obtained from EHR data. These models will include a fixed binary effect for intervention condition (baseline vs. intervention/maintenance), a fixed effect for baseline outcome rate for each specific clinic as a covariate, other patient- or clinic-level covariates as appropriate, fixed effects for health system (4 *df* for five health systems), and a random effect for the clinic to account for clustering. The primary outcome will be coded as 1 if the patient's CD status was documented (i.e., the CD field is not blank in the EHR) and 0 otherwise.

Secondary analyses will include a third level of the intervention variable (i.e., splitting intervention from maintenance periods) to determine whether intervention effects persist over time after practice facilitation ends. This is particularly important given the study's focus on sustainability of disability documentation practices. Goodness-of-fit statistics and model fit diagnostics will be used to assess modeling assumptions, with appropriate remedial measures taken if strongly violated (e.g., transformations of covariates). Hypothesis tests will be two-sided with α = .05 and *p* values reported. Other quantitative outcomes will be derived from surveys and will be analyzed descriptively.

Given the quasi-experimental stepped wedge design, we will conduct several analyses to address potential threats to internal validity from the nonrandomized implementation. We will assess the baseline equivalence of early versus late-adopting clinics on key patient characteristics (age, race, ethnicity, primary language, insurance status) and clinic characteristics (size, urban vs. safety-net setting, baseline documentation rates, participation in other quality improvement initiatives). We will include calendar time as a covariate in our models to account for secular trends across the study period. All statistical analyses will be performed using SAS (Version 9.4; SAS Institute Inc., 2013) or R (R Foundation for Statistical Computing).

### Qualitative Analysis

For all qualitative data, we will employ qualitative content analysis throughout our data collection and analyses (Hsieh & Shannon, 2005). All interview transcripts will be imported into Atlas.ti (Version 23) to assist with sorting the texts for analysis. We will use an inductive coding process in which two or more study team members will independently develop codes and associated definitions from reading the transcripts. The team will meet and discuss their respective codes to develop a consolidated codebook. The study team will independently apply the codebook to the next set of transcripts and then meet and reconcile their codebooks and coded data. This process will continue until a final codebook is agreed upon. This codebook will be applied to the remaining transcripts, with at least 20% of the transcripts coded by two team members to ensure consistency of the application of the codebook. Discrepancies will be reconciled through consensus. If the coders are unable to reach consensus, a third team member will make the final coding decision. Data will be analyzed within and across clinics to identify major themes related to the adoption, implementation, effectiveness, and maintenance of disability documentation and accommodation provision practices. We will identify barriers, facilitators, adaptations, and contextual factors that influence implementation.

### Mixed-Methods Integration

Once we have preliminary findings from both the quantitative and qualitative analyses, we will integrate the data using a concurrent mixed-methods approach. We will create joint displays with quantitative outcomes (e.g., documentation rates, accommodation provision rates) with qualitative themes (e.g., barriers, facilitators, adaptations, contextual factors) for each clinic and across clinics. We will look for instances in which quantitative and qualitative findings converge, diverge, or expand upon each other to identify patterns that provide additional insights into the results. Based on the integrated findings, we will modify and expand the implementation guide to reflect lessons learned about effective implementation approaches, common barriers and solutions, and contextual factors that facilitate or hinder successful adoption and maintenance of CD documentation and accommodation provision practices in primary care settings.

## PRECIS-2-PS Assessment

To systematically assess how pragmatic our study design is, we use the PRECIS-2-PS tool (Norton et al., 2021). The PRECIS-2-PS is a self-assessment tool for researchers conducting trials that evaluate provider-focused implementation strategies. It extends the original PRECIS-2 framework and helps researchers align study design decisions with the study's intended purpose, ensuring that results are applicable to the real world if pragmatic or provide mechanistic understanding if explanatory.

The PRECIS-2-PS evaluates nine domains of research design: eligibility, recruitment, setting, implementation resources, flexibility of provider strategies, flexibility of intervention, data collection, primary outcome, and primary analysis. Each domain is scored on a 1–5 scale, where higher scores indicate more pragmatic choices (e.g., usual care settings, minimal additional resources, clinically meaningful outcomes) and lower scores indicate more explanatory features (e.g., highly selected participants, intensive resources, mechanistic outcomes). The scores are displayed on a wheel diagram (see Figure 2). Below is the PRECIS-2-PS scores for the DECA study and our scoring rationale for each domain.

**Figure 2.**
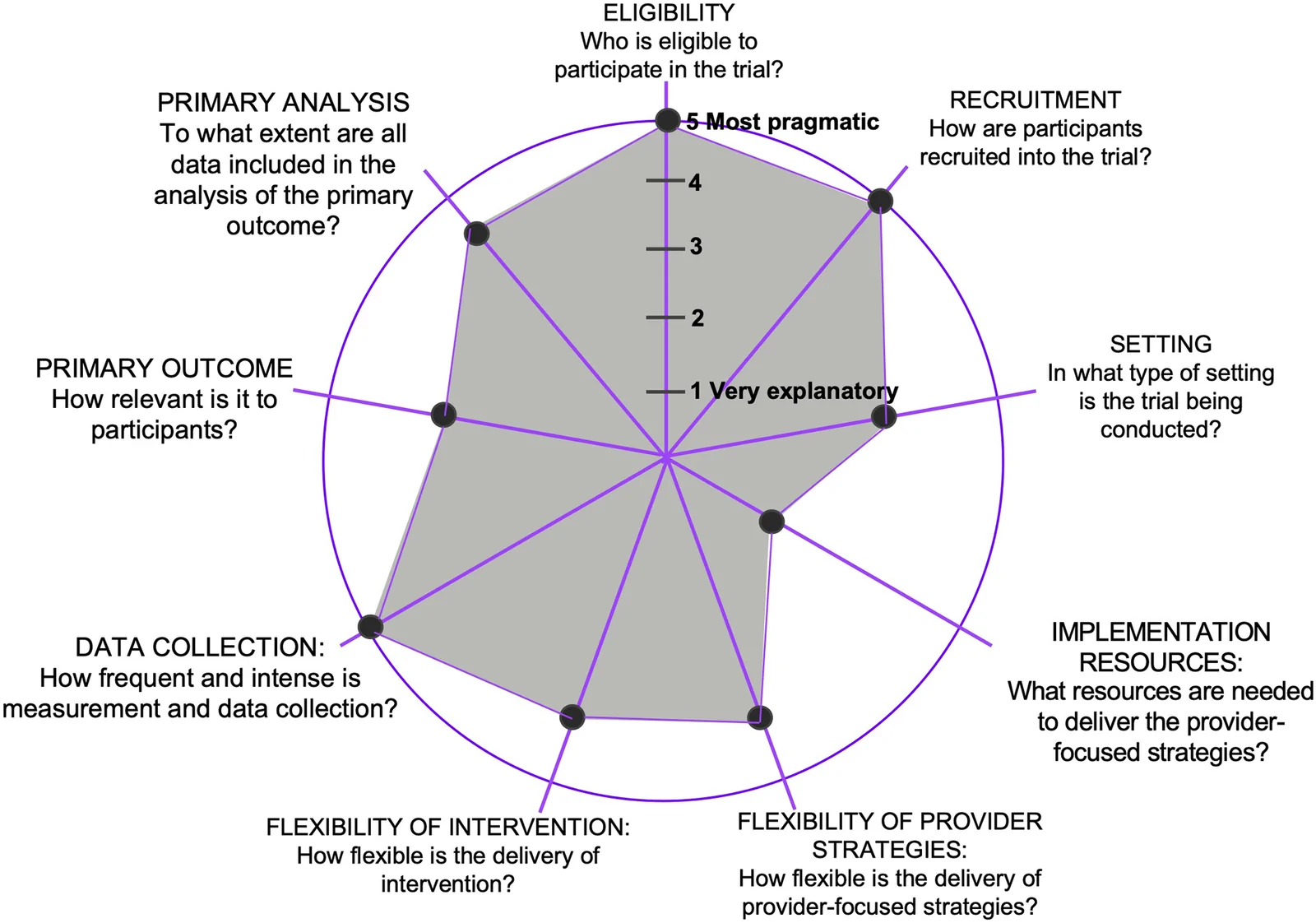
Pragmatic Explanatory Continuum Indicator Summary-2.

### Eligibility: Score 5

The intervention targets staff and clinicians within participating primary care clinics. The Eligibility domain asks the extent to which the health care professionals in the trial are similar to those in usual care. We rated this as highly pragmatic (5) because all clinicians, staff, and clinic leaders employed in participating clinics are eligible to participate in the trial, with no exclusion criteria beyond being 18 years or older (a criterion we do not anticipate excluding any health care professionals). This inclusive approach ensures that our findings reflect the full range of health care team members who would implement CD documentation and providing accommodations in real-world primary care settings.

### Recruitment: Score 5

Participants include any health care team members (e.g., physicians, medical assistants, nurses, registration staff) employed in participating primary care clinics. The Recruitment domain assesses the degree of effort made to recruit health care team members compared to usual care settings. We rated this as highly pragmatic (5) because no special recruitment efforts beyond usual care are made. Health care team members participate as part of their routine clinic responsibilities. Each clinic selected the team members who will participate in practice facilitation sessions based on their roles and responsibilities. For qualitative data collection (leadership interviews, staff and clinician focus groups), there are no exclusion criteria, although we anticipate that more engaged team members will volunteer. Recruitment for these optional study activities will occur through regular mass clinic e-mails.

### Setting: Score 3

The Setting domain asks how different the health care settings are compared to usual care settings. We rated this as moderately pragmatic (3). On the pragmatic side, participating sites have engaged leadership who proactively chose to adopt CD documentation initiatives. However, several factors reduce pragmatism. All participating clinics are affiliated with academic medical centers and located in urban settings, limiting generalizability to community-based or rural primary care practices. Additionally, all sites use the Epic EHR system and have electronic data warehouses—requirements necessary for feasibility and consistent data collection for the study. However, Epic is the most widely used EHR in the United States, particularly among health systems likely to be early adopters of disability documentation initiatives, making our findings applicable to a substantial portion of the target population (Chishtie et al., 2023). We did not score lower than 3 given the diversity across clinic types (e.g., geriatric clinics, specialized clinics, safety-net systems).

### Implementation Resources: Score 2

The Implementation Resources domain assesses how different the resources needed to support the provider-focused strategies are from those readily available in usual care. We rated this as explanatory (2) because practice facilitation is provided entirely by the research team and is not currently available at all participating sites as part of usual care. We did not score this domain lower for two reasons. First, practice facilitation is an established and commonly used method in primary care settings to implement and sustain practice changes, making it a realistic implementation strategy (not a novel research intervention; Baskerville et al., 2012). Second, many participating sites had previously used practice facilitation for other quality improvement initiatives, demonstrating feasibility and familiarity in these settings. However, for translating findings to practice, clinics would need to either employ practice facilitators or adapt the implementation guide for more self-directed use.

### Flexibility of Provider-Focused Strategies: Score 4

The Flexibility of Provider Strategies domain refers to the flexibility in how provider-focused strategies are delivered in the study compared to usual care. Practice facilitation is our provider-focused strategy. We rated this as highly pragmatic (4) because the practice facilitation sessions are highly flexible and tailored to each clinic's needs. The practice facilitator works with each clinic to assess their unique barriers, assists in problem solving, and ensures that CD documentation and accommodation provision processes are adapted to local workflows, resources, and clinic culture. We did not score this as fully pragmatic because we require clinics to meet with the practice facilitator once monthly for 9 months. However, within these meetings, the content, focus, and problem-solving approaches are driven by each clinic's needs and context.

### Flexibility of Intervention: Score 4

This domain refers to the flexibility in how the intervention is delivered by health care team members compared to usual care. We rated this as highly pragmatic (4) because clinics retain flexibility in (a) how they collect disability information, (b) how they design disability data fields and where they are located in the EHR to fit local workflows, (c) what additional disability questions they collect beyond the two required CD questions, and (d) how they provide accommodations (processes, workflows, staff responsibilities). This flexibility ensures that the intervention can be adapted to diverse clinic contexts and resources. We did not score this as fully pragmatic because we require all clinics to collect the two CD questions and to enter the information into the EHR.

### Data Collection: Score 5

The Data Collection domain refers to the frequency and intensity of measurement and data collection throughout the trial compared to what would be expected in usual care. We rated this as highly pragmatic (5) because the primary outcome, CD status documentation, is extracted from EHR data generated through routine care. All participating sites have leadership support to document CD and accommodation needs independent of the study; as such, this data collection is part of the care provided rather than done solely for research purposes. Sites retain flexibility to adjust the timing, frequency, and method of data collection according to their local workflows. No additional data collection visits, assessments, or procedures beyond routine care are required for the primary outcome.

### Primary Outcome: Score 3

This domain reflects the extent to which the primary outcome is of interest and importance to health care teams and patients. Norton et al. (2021) state that the most pragmatic outcomes are of obvious interest to stakeholders (such as quality of life or patient satisfaction), whereas explanatory outcomes are process-oriented measures. We rated this as moderately pragmatic (3). Our primary outcome, documentation of disability and accommodation needs in the EHR, is process oriented rather than patient centered, which reduces pragmatism. However, this process outcome has been explicitly identified as a critical need by multiple stakeholder groups, including disability advocates, clinicians, patients, health system leaders, and policy makers (Morris, Lockhart, et al., 2021). Furthermore, disability documentation is a necessary prerequisite for the patient-centered outcomes stakeholders ultimately care about (receiving appropriate accommodations, improved communication, equitable care). For this study, measuring whether the intervention is implemented (documentation) is both appropriate and meaningful to stakeholders.

### Primary Analysis: Score 4

This domain relates to the extent to which all data are included in the analysis of the primary outcome, compared to more restrictive explanatory analyses. We rated this as highly pragmatic (4) because we will include all patient data for adults 18 years and older who had at least one visit during the 15-month EHR data collection period at participating study clinics. We will not exclude patients based on diagnosis, visit type, insurance status, or any other characteristic. We will use an intent-to-treat approach at the clinic level, analyzing documentation rates regardless of individual clinic implementation fidelity or staff adherence to the CD documentation process. This means that clinics are analyzed according to their assigned study period, even if they struggled with implementation or experienced barriers. We did not score this as fully pragmatic because we are limited to analyzing data from clinics that completed their EHR builds. However, this reflects the real-world requirement that systems must have basic infrastructure to implement CD documentation.

### Summary of PRECIS-2-PS Assessment

The DECA study demonstrates strong overall pragmatism with intentional trade-offs that balance real-world applicability with scientific rigor. The design is highly pragmatic in eligibility (5), recruitment (5), data collection (5), primary analysis (4), flexibility of provider strategies (4), and flexibility of intervention (4), ensuring that findings are applicable to the full range of health care team members and patients seen in primary care settings. The design is moderately pragmatic in setting (3) and primary outcome (3), reflecting necessary structure to ensure study feasibility and rigor. The least pragmatic domain is implementation resources (2) because practice facilitation requires dedicated resources not currently available at all sites. This trade-off was intentional and necessary. We hypothesized that without practice facilitation support from the study, clinics would be unlikely to successfully adopt CD documentation practices.

Overall, the PRECIS-2-PS profile demonstrates that the DECA study prioritizes external validity and real-world applicability while maintaining sufficient rigor to draw conclusions about the effectiveness of practice facilitation as an implementation strategy. The pragmatic design choices ensure that findings will be actionable for clinics considering CD documentation and accommodation provision initiatives and provide realistic expectations about the resources and flexibility needed for successful implementation.

## Discussion

The DECA study is the first multisite pragmatic trial evaluating the implementation of documenting CD status and accommodation needs in the EHR and using the information to provide CD accommodations in primary care settings. It is ultimately a system-level intervention targeting the communication environment in health care for people with CDs. Pragmatic trials are designed to test interventions under real-world conditions that closely mirror usual care, prioritizing external validity and applicability over the tightly controlled conditions of explanatory trials. This approach is essential for implementation science studies, which seeks to understand not whether an intervention works under ideal circumstances but how to successfully integrate evidence-based practices into everyday clinical care (Curran et al., 2012). As demonstrated through our PRECIS-2-PS assessment, the study design is moderate to highly pragmatic across most domains. We made intentional design trade-offs that balance real-world applicability with scientific rigor. This pragmatic approach ensures that our findings will be directly applicable to health systems and primary care clinics considering disability documentation initiatives, while providing realistic expectations about the implementation process, barriers, and resources needed for success.

### Improving the Environment of Health Care for People With CDs

This study addresses the communication environment in health care at a health system level. Environmental factors are substantial contributors to effective communication for people with CDs, but addressing them can be challenging for CSD clinicians (Kagan et al., 2024; O'Halloran et al., 2008, 2011, 2012; O'Halloran & Larkins, 2008; Simmons-Mackie, 2013). We anticipate that this study will have important downstream implications for CSD clinicians. First, documenting CD status and providing accommodations in the EHR provide a system-level valuation of the importance of effective communication in achieving high-quality health care outcomes. Although this study will focus on hearing amplifiers and communication boards, it provides a template for relaying other accommodation needs through the EHR, such as communication strategies. Second, these practices set a standard that all health care team members are responsible for effective communication. This is important in the context of current practices where CSD clinicians struggle to enlist health care team members in implementing accommodations for patients with CDs (Barnard et al., 2022a, 2022b; Chang et al., 2018). Realizing this practice expands the CSD clinicians' reach in more effectively addressing environmental factors impacting effective communication within health care settings.

### Advancing Pragmatic Research in CSD

This article provides an example for researchers in CSD interested in conducting pragmatic implementation trials. Historically, much of CSD research has focused on efficacy studies conducted under controlled conditions, which, while essential for establishing that interventions work, often have limited applicability to real-world clinical settings (Douglas et al., 2022). There is a critical need for the field to grow in conducting pragmatic implementation research that bridges the research-to-practice gap (National Institute on Deafness and Communication Disorders, 2017, 2023). Pragmatic trials test interventions in the messy, complex environments where they will ultimately be used, accounting for the contextual factors, resource constraints, and workflow challenges that influence implementation success (Holtrop & Glasgow, 2020). By transparently documenting our design decisions, the trade-offs we encountered, and our use of frameworks such as PRECIS-2-PS and RE-AIM, we aim to provide practical guidance for CSD researchers embarking on similar implementation studies. As the field increasingly recognizes the importance of demonstrating not just efficacy but also real-world effectiveness and implementation, pragmatic trial designs will be essential for generating evidence for clinicians, health systems, and policymakers.

### Lessons From Adapting to External Context

Our shift from a randomized to quasi-experimental stepped wedge design illustrates a critical lesson in pragmatic research: External contextual factors can have significant impact on study execution. In our case, the health care policy landscape changed substantially during the study period. When the Office of the National Coordinator released disability status data element standards and the Joint Commission introduced a new health equity certification in 2023 (Assistant Secretary for Technology Policy, 2023; The Joint Commission, 2023), our participating health systems' motivations increased, but timelines shifted. A core component of successful pragmatic research is understanding the motivations of participating collaborators and adapting design decisions to balance pragmatism with internal validity. By allowing site flexibility in implementation timing, we can test the intervention under conditions that reflect how health systems make strategic decisions about adopting new documentation practices—in response to policy and accreditation requirements rather than random assignment.

### Strengths of the Study Design

Several design features strengthen the DECA study. First, the implementation guide was created through implementation mapping (Oshita et al., 2025), an evidence-based, stakeholder-informed method that engaged disability experts, researchers, health system stakeholders, and people with disabilities (Fernandez et al., 2019). This collaborative process resulted in a comprehensive guide that addresses real-world barriers and is grounded in the perspectives of those most affected by disability documentation practices. The guide is flexible and allows clinics to adapt the processes to their local contexts, which will assist with sustainable implementation. Second, our use of EHR data for the primary outcome enhances both pragmatism and scalability. Documentation rates extracted from routine clinical data require no additional data collection burden on clinics or patients, can be monitored continuously, and reflect actual practice rather than reported behavior. Third, the mixed-methods design provides rich data on not just whether practice facilitation improves documentation and accommodation provision rates but also how implementation unfolds in the primary care context, what barriers emerge, what adaptations clinics make, and what factors predict successful implementation and maintenance. This information is essential for translating findings to other primary care clinics and for refining the implementation guide based on lessons learned. Fourth, we are conducting the study in 10 clinics across five health systems. Testing the intervention across multiple sites allows us to identify universal implementation principles and context-specific factors that influence success.

### Limitations and Considerations for Interpretation

Several limitations will need to be considered when interpreting the study's findings. The most significant limitation is the lack of randomization in determining when clinics begin the intervention period. Moving from a randomized to quasi-experimental design introduces potential selection bias (early vs. late adopters may differ systematically) and confounding by time (secular trends or external events may influence outcomes independent of the intervention). We will address these threats through assessing the baseline characteristics of clinics, statistically controlling for clinic and patient characteristics and thoroughly documenting contextual factors. The pragmatic justification for this trade-off is that health systems and clinics in real-world settings do not randomly decide when to implement new practices; rather, they make strategic decisions based on readiness, resources, and institutional priorities. Our findings will reflect these realistic conditions.

Second, the study's health systems are predominantly urban, affiliated with academic medical centers, and all use the Epic EHR system. This limits generalizability to rural primary care practices, community-based health centers, and practices using other EHR systems. While Epic is the most widely used EHR in the United States and our participating systems include safety-net settings, the lack of rural and smaller primary care clinic representation is a gap. Additionally, our focus on early adopters—health systems with engaged leadership, established quality improvement infrastructure, and resources to participate in research—may inflate implementation outcomes and limit applicability to less-resourced settings. We will collect data on health system and clinic characteristics and integrate these characteristics when interpreting implementation findings.

A third limitation is that practice facilitation is resource intensive, requiring a trained facilitator and clinic team time for monthly meetings over 9 months. While this approach may not be feasible for all clinics, particularly smaller practices or those with limited resources for quality improvement initiatives, it is still critical to determine whether practice facilitation is effective for supporting disability documentation.

Fourth, we will not conduct an economic evaluation due to time and resource constraints in the project. Understanding the costs associated with practice facilitation, EHR build development, accommodation purchases, and staff time, as well as potential cost savings from improved care quality and reduced disparities, would provide critical information for health systems and clinics making implementation decisions. Future research should include comprehensive economic evaluations to inform cost-effectiveness and return on investment.

### Future Directions

The DECA study paves the way for several important avenues for future research. First, as CD documentation becomes more widespread in response to policy requirements, health systems' and clinics' interest and adoption will likely increase. Future studies should include rural practices, smaller health systems, community health centers, and practices using diverse EHR platforms to enhance generalizability and identify how implementation strategies may need to be adapted for different settings and resource levels.

Second, future research should test adaptive implementation designs where implementation strategy intensity is escalated based on clinic performance. For example, clinics might begin with self-directed use of the implementation guide, with practice facilitation provided only to clinics that struggle to achieve adequate documentation rates. This stepped approach would make implementation support more efficient and scalable while still providing intensive support to clinics that need it.

Third, future research should expand beyond CD to include all disability types. While we focused on CD as an initial starting point, health systems are legally required to provide accommodations to all patients with disabilities, which represents 27% of the U.S. adult population (Varadaraj et al., 2021). Starting with CD and communication accommodations allowed us to develop and test our implementation approach with a more focused scope. However, broadening documentation to all disability types could increase adoption, as it applies to a greater proportion of health systems' and clinics' patient populations. The lessons learned from this study will have important implications for future research addressing general disability documentation and accommodation provision.

Fourth, future research should examine how disability documentation practices are maintained and sustained over time, particularly after practice facilitation or other implementation support ends. If practice facilitation proves effective, we will need to determine how to scale the intervention, or at least its essential components (such as structured, regular meetings that assist clinics in problem-solving challenges that arise), in ways that are feasible for health systems and clinics.

Fifth, as health systems and clinics begin routinely documenting disability status, research and quality improvement initiatives should shift toward using disability status data meaningfully. This includes tracking the quality of care provided to patients with disabilities to identify opportunities for improvement. Additionally, these data can inform processes and systems for providing accommodations. For example, if a clinic identifies a high proportion of patients with a specific disability type, such as blindness, they can invest in accommodations tailored to that population's needs. Linking routine documentation to outcomes meaningful to leadership will be essential for demonstrating the value of disability documentation initiatives and sustaining institutional commitment. This evolution from implementation to effectiveness and quality improvement research will be critical for translating disability documentation into meaningful improvements in health care quality.

## Conclusions

The DECA study will provide critical evidence on how to support primary care clinics in implementing systematic CD documentation and provision of accommodations. This system-level intervention addresses the environment of communication in health care, thus embedding the importance of effective communication into routine care delivery. By employing a pragmatic design that balances real-world applicability with scientific rigor, we will ensure that findings will be actionable for health systems navigating the complex process of integrating disability documentation into routine care. Our transparent discussion of design trade-offs, particularly the shift from randomized to quasi-experimental design in response to changing policy contexts, provides practical guidance for other CSD implementation researchers facing similar challenges. As health care systems and clinics increasingly recognize the imperative to improve the quality of care for people with CD, the DECA study offers an evidence-based roadmap for a foundational step: knowing who has a CD and what accommodations they need. This knowledge is essential for providing high-quality, accessible, patient-centered care for the 14% of adults with CD and the broader disability community.

## Data Availability Statement

This article describes a study protocol and does not contain any data. Data sharing details are not applicable at this stage, but the researchers commit to making data available in accordance with ethical guidelines and a specific data sharing plan once the study outlined in this protocol is conducted.

## Supplementary Material

10.1044/2026_JSLHR-26-00080SMS1 Supplemental Material S1 Standards for Reporting Implementation Studies: the StaRI checklist for completion.

## Notes

**Publisher Note:** This article is part of the Forum: Research Symposium on Clinical Trials Conducted With Adult Populations.
